# Characterization of the Demographics and Psychiatric Co-Morbidites Among Clients of a Human Rights Clinic in Miami-Dade County, Florida, United States

**DOI:** 10.7759/cureus.8944

**Published:** 2020-07-01

**Authors:** Samuel Kareff, Chanelle Diaz, Amy Zeigler, J. Grey Faulkenberry, Brigitte F Utter, Cecily M Barber, Stephen Symes

**Affiliations:** 1 Internal Medicine, MedStar Georgetown University Hospital, Washington, D.C., USA; 2 Internal Medicine, Montefiore Medical Center, New York, USA; 3 Emergency Medicine, University of Pittsburgh, Pittsburgh, USA; 4 Biomedical and Health Informatics, Children's Hospital of Philadelphia, Philadelphia, USA; 5 Family Medicine, Denver Health and Hospitals, Denver, USA; 6 Obstetrics and Gynecology, Thomas Jefferson University, Philadelphia, USA; 7 Division of Infectious Diseases, University of Miami Miller School of Medicine, Miami, USA

**Keywords:** asylum-seekers, refugee health, major depressive disorder, post-traumatic stress disorder

## Abstract

Miami-Dade County (MDC) represents a major port of entry for people seeking asylum in the United States, and few studies have systematically evaluated the demographic characteristics of this vulnerable population. Moreover, while the burden of post-traumatic stress disorder (PTSD) and major depressive disorder (MDD) are thought to be higher in this population, the prevalence of these psychiatric conditions in our community is unknown. An analysis of demographics and psychiatric co-morbidities of the Human Rights Clinic (HRC) of Miami’s 93 clients between 2010 and 2015 was conducted. The HRC cohort had the following characteristics: median age of 30 years, 52% female, 46% male, 2% transgender or intersex, and 88% originating from Latin America and the Caribbean. The prevalence of PTSD was 67% and MDD was 53% in the HRC population. We conclude that the mental health burden in asylum-seekers in MDC is alarmingly high and that healthcare providers should remain keenly attentive to the unique needs of this population.

## Introduction

Objectives

1) To illustrate key epidemiologic data of the Human Rights Clinic (HRC) of Miami asylum-seeking population.

2) To compare the demographics of the HRC cohort with those of Miami-Dade County (MDC).

3) To quantify the prevalence of post-traumatic stress disorder (PTSD) and major depressive disorder (MDD) through validated screening tools and compare to similar populations locally, nationally, and globally.

Background

Every year, tens of thousands of individuals arrive to the United States seeking various forms of asylum from torture and other forms of maltreatment suffered in their home countries [1]. MDC represents a major port of entry for asylum-seekers, especially those originating from Latin America and the Caribbean. While rates of PTSD and MDD are higher in populations exposed to trauma such as asylum-seekers, it is not known what the prevalence of these psychiatric conditions is among asylum-seekers residing in MDC [2-4]. Although there have been previous attempts at characterizing the medical status of asylum-seekers in MDC, little is known about the specific populations that are most likely to settle in MDC [5]. Moreover, it is difficult to project their individual needs or the impact to the local healthcare system without a demographic understanding of this population.

Purpose

The HRC, established in 2010, is an independent organization of medical students, residents, and attending physicians who complete medical and/or psychiatric examinations free of charge for individuals seeking asylum and other forms of immigration relief in South Florida. The healthcare provider’s systematic documentation of evidence of torture and/or human rights abuses, either physical or psychological, is then incorporated into a legal affidavit that becomes part of an applicant’s official application for asylum. This affidavit can play a crucial role in the subsequent legal decisions made about a case. It has been demonstrated previously that with the assistance of corroborating physicians, applicants who provide courts with medical affidavits are granted asylum at rates of up to 89%, more than double the national asylum approval rate of 37.5% and higher than the average asylum approval rate in MDC which has been estimated as low as 15.6% [6-7]. As of December 2015, HRC had evaluated 93 clients requesting legal affidavits to accompany asylum applications or other immigration proceedings petitioning legal protection within the United States. Thus, the main purpose of this study is to provide a descriptive analysis of the population served by HRC with an emphasis on the mental health needs unique to this population.

## Materials and methods

Participants

The HRC receives evaluation requests from a variety of community partners, including national organizations and private law firms, in the MDC metropolitan area. This analysis included all clients who received an evaluation from HRC between May 2010 and December 2015. Clients of the clinic are generally adults aged 18 and over who have requested the services of the clinic through their legal representation. Clients or their legal guardians provide informed, written consent to participate in clinic activities. The HRC clients are interviewed in physician or legal offices, free clinics, or immigration detention centers. 

Data collection

The HRC student coordinators maintain a password-protected, secured, and de-identified database including information collected prior to and during evaluations. Such information includes basic demographics, type of immigration case, referral source, reasons for seeking asylum, history of torture/human rights abuses reported, and case outcomes.

Measures and analyses

A retrospective, descriptive analysis of the HRC database was performed. Demographics are reported as percentages, frequency distributions, and central tendencies. Prevalence of PTSD and MDD were operationalized by positive screening results on the PLC-C Civilian Screening Tool, a validated screening instrument for PTSD from the United States Veterans Administration, and the PHQ-9 or HAM-D, validated screening tools for MDD that utilize DSM-IV depression diagnostic criteria [8-11]. Stata (version 12.0, StataCorp, College Station, TX) was used for all analyses.

Ethics approval

The Office of the Human Research Protection Program of the University of Miami Miller School of Medicine considered this secondary data analysis of previously collected, de-identified data exempt from additional review.

## Results

Some key demographic data points regarding the HRC cohort, along with parameters from MDC at large, are presented in Table 1 [12]. Some 93 clients were evaluated between 2010 and 2015. Specifically, the cohort’s average age was 30 years. The proportion of women was 51% (n=47), with a remaining 47% male (n=44) and 2% transgender or intersex (n=2). Some 88% (n=82) of clients were from Latin American or Caribbean countries, with 47% (n=44) nationals of Central American countries. 

**Table 1:** Key demographic characteristics of 93 clients served by the HRC of Miami compared to those of MDC. HRC, Human Rights Clinic; MDC, Miami-Dade County

Parameter	HRC cohort	MDC
N	93	2,662,874
			Median Age, Years (SD)	30 (13.4)	39.3 (N/A)
Gender (%)					
Female	50.5	51.4
Male	47.3	48.6
Trans/Intersex	2.2	N/A
Preferred Language (%)		
English	14	27.7
Other than English	86	72.3
Spanish	56	N/A
Haitian Creole	26	N/A
Other	4	N/A
Region of Birth of Foreign Born (%)		
Latin America and the Caribbean	88.2	92.7
Not Latin American and the Caribbean	11.8	7.3
Asia	6.5	2.8
Europe	3.2	3.5
Africa	2.2	1

Some 67% (n=62) and 53% (n=49) of the HRC cohort screened positive for PTSD and MDD, respectively. Furthermore, 82% (n=76) reported history of torture and/or human rights abuses including physical, psychological, sexual, or multiple forms of trauma. The most common forms of torture were beatings and/or whippings (67%, n=62) and cutting/stabbing with sharp instruments (15%, n=14). Other forms of torture reported by HRC clients included, but were not limited to, heat and chemical burns, electric shock, suffocation and/or drowning, suspension, and witnessed torture and killings. Some 20% (n=19) of clients were fleeing gang or drug trafficking-related violence, including 50% (n=12) of unaccompanied minors.

Rates of intimate partner violence were 38% (n=18) among women and 100% (n=2) among transgender clients. Additionally, 66% (n=31) of women and 50% (n=1) of transgender clients reported sexual violence. Rates of reported adverse childhood experiences such as domestic or sexual abuse were 33% (n=31) among all clients, and 71% (n=17) among unaccompanied minors.

## Discussion

Demographically, the HRC cohort mirrored the MDC population at large in terms of median age (30 and 39.9, respectively), gender (51 and 51.4% female, respectively), and region of origin (88.2 and 92.7% from Latin American and the Caribbean, respectively). This observation could be explained by familial, cultural, economic, and political ties between the local setting and asylum-seekers’ native countries. Such a “pull” represents one means by which these populations can cope with the stress of acculturating to a new milieu [13]. Notably, the most common countries of origin of the HRC cohort differed drastically from the general population of asylum seekers in the United States with more Asian and African countries represented nationally (see Table 2) [14].

**Table 2:** The five most common countries of origin for HRC and United States asylum-seekers. HRC, Human Rights Clinic

HRC (2010-2015) (total % originating)	United States (2015) (total % originating)
1. Haiti (26.8%)	1. Myanmar (Burma) (26.3%)
2. Honduras (24.7%)	2. Iraq (18.1%)
3. Guatemala (9.7%)	3. Somalia (12.7%)
4. El Salvador (7.5%)	4. Democratic Republic of Congo (11.3%)
5. Nicaragua (4.3%)	5. Bhutan (8.3%)

New contributions to the literature

**Figure 1:**
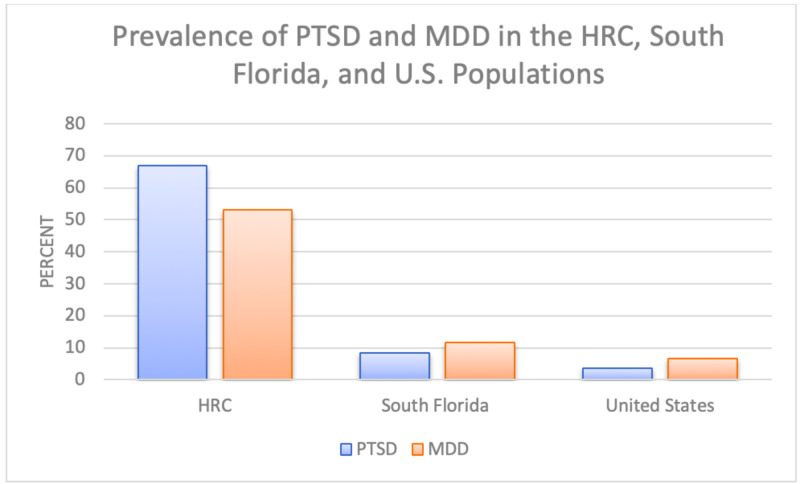
Prevalence of PTSD and MDD in various North American cohorts. PTSD, post-traumatic stress disorder; MDD, major depressive disorder

Conversely, the HRC cohort displayed a high prevalence of PTSD (67%) and MDD (53%). These findings are consistent with other studies of asylum-seekers and refugees around the world. For example, Bosnian refugees who resettled in Austria and Australia were also found to have higher prevalences of PTSD, depressive, and anxiety symptoms upon arrival [15]. Furthermore, 36.1% of various African refugees in Hong Kong and 38.3% of Somali refugees in Ethiopia were found to screen positive for depressive symptoms [16-17]. Moreover, the HRC cohort’s prevalences of these mental health conditions are much higher than estimates for the general South Florida population (8.4% PTSD and 11.6% MDD) or United States (3.5% PTSD and 6.7% MDD) populations (see Figure 1) [18-19].

Notably, 82% of the HRC cohort reported episodes of torture and/or human rights abuses; prevalence estimates of torture history vary from 6.6% at an urban primary care clinic to 11.5% in an urban emergency department cohort, and often go unreported [20-21]. Furthermore, the asylum-seekers’ legal affidavits reveal a wide variety of types of abuse suffered including persecution of marginalized political sects in Ethiopia, gang violence in Honduras and El Salvador, and sexual assault in Haiti and the Bahamas.

Limitations

 To the authors’ knowledge, these estimates provide the first demographic and medical evidence regarding the unique cohort of asylum-seekers who settle in MDC. However, a significant limitation to our findings is the lack of validation of the PLC-C, PHQ-9, and HAM-D screening tools in our specific study population, as these instruments have only been previously validated for primary care settings. Another argument is that the data we have presented herein might resemble a convenience sample due to their physical location during interview; detained individuals constitute a significant minority of our study population (14%, n=13).

An additional limitation of our data is its focus on the psychiatric comorbidities exhibited by the HRC cohort upon the time of medical/psychiatric evaluation. Further research is needed to clarify acute medical conditions also experienced by this vulnerable population, including injuries related to instances of trauma and torture. Screening for other chronic medical conditions, including overweight, hypertension, diabetes mellitus, and chronic pain for which this population may be at greater risk, would also be of benefit for elucidating this population’s medical burden.

Implications for public health practice

 The data discussed in this analysis can have important implications for local officials, public health officers, and healthcare providers. First and foremost, these findings highlight that additional resources should be dedicated to assist these clients with their large burden of psychiatric comorbidities. Currently, the Florida Department of Health in MDC operates a Refugee Health Assessment Program (RHAP) in which a “mental health evaluation and referral” process is available to refugees, parolees, and asylum-seekers who lawfully enter into the United States [22]. It would benefit the HRC cohort as well as larger populations, including Cuban and Haitian entrants, for the RHAP to develop a more robust mental health arm capable of reaching populations in need. Secondly, healthcare providers practicing in MDC, as well as other major ports of entry, should have a greater knowledge and understanding of the unique mental health needs of asylum-seekers settling within the United States, and should screen these patients for histories of trauma and torture. This is essential given that evidence suggests that stigma and discrimination targeting these populations can lead to a poorer quality of care [23]. 

## Conclusions

Asylum-seekers in MDC mirror the demographics of the county at large but appear to have a much larger burden of psychiatric co-morbidities including PTSD and MDD. Moreover, the majority of these individuals has faced episodes of torture and/or human rights abuses in their countries of origin. These results suggest that local public health authorities and healthcare providers design tailored outreach programming to target this vulnerable population and ensure access to needed services.
